# Exercise‐Induced Myonectin Protects Against Cerebral Ischemia–Reperfusion Injury

**DOI:** 10.1002/cns.71137

**Published:** 2026-09-11

**Authors:** Jing Zhang, Shengle Chen, Jingyu Liu, Xin Qi, Xiaoming Zhang, Peng Lin, Dezhang Huang, Jing Li

**Affiliations:** ^1^ Key Laboratory of Marine Drugs, Chinese Ministry of Education, School of Medicine and Pharmacy Ocean University of China, Laboratory for Marine Drugs and Bioproducts of Qingdao National, Laboratory for Marine Science and Technology Qingdao China; ^2^ Department of Neurosurgery, Qilu Hospital (Qingdao), Cheeloo College of Medicine Shandong University Qingdao Shandong China

**Keywords:** exercise, ischemia–reperfusion, myonectin, neuroprotection, PI3K/Akt/mTOR

## Abstract

**Background:**

There exists muscle–brain signaling via myokines. However, not all members of myokines have been thoroughly studied. Here, we identify myonectin, an exercise‐induced myokine, as a new regulator of cerebral ischemia–reperfusion (I/R) injury.

**Methods:**

Cerebral ischemia was induced by middle cerebral artery occlusion (MCAO/R) in wild‐type mice and skeletal muscle‐specific myonectin‐knockout mice. Laser speckle contrast imaging (LSCI) and TTC staining were used to assess cerebral injury. HT22 and BV2 cells were utilized to mimic I/R–associated cellular damage using oxygen–glucose deprivation and reoxygenation (OGD/R). Intravenous recombinant myonectin was administered to assess therapeutic effects on I/R injury. Immunohistochemistry, TUNEL, ELISA, and Western blotting assays were performed to detect morphological and molecular changes in cells and tissues.

**Results:**

Exercise elevated serum and brain myonectin and reduced infarct size and neuronal damage in wild‐type but not myonectin–deficient mice after MCAO/R. Systemic myonectin administration significantly protected against cerebral I/R injury. Mechanically, myonectin exerted neuroprotective effects by suppressing excessive autophagic apoptosis and microglia‐mediated neuroinflammation thorough activation of the PI3K/Akt/mTOR pathway.

**Conclusion:**

Skeletal muscle‐derived myonectin protects the brain from I/R injury, contributing to exercise benefits via muscle–brain communication. Myonectin may serve as a promising therapeutic agent against cerebral I/R injury.

## Graphical abstract

Exercise‐induced myonectin protects against cerebral ischemia–reperfusion injury. Myonectin knockout abolishes exercise benefits, whereas recombinant administration reduces cerebral infarct size. Mechanistically, myonectin activates PI3K/AKT/mTOR signaling, thereby suppressing excessive neuronal autophagy and microglial‐driven neuroinflammation.

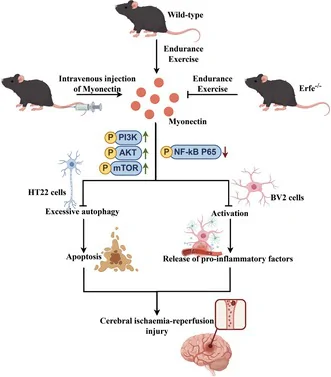

**Abbreviations**
3‐MA: 3‐methyladenine
CBF: cerebral blood flow
Erfe: erythroferrone
I/R: cerebral ischemia/reperfusion
LY294002: LY
MCAO/R: middle cerebral artery occlusion/reperfusion
Myonectin: myo
NeuN: neuronal nuclei
OGD/R: oxygen–glucose deprivation/reoxygenation
Rapamycin: ra
ROS: reactive oxygen species
TTC: 2,3,5‐triphenyltetrazolium chloride

## 1 Introduction

Stroke continues to represent a substantial global health burden, ranking among the primary causes of death and persistent disability worldwide. Globally, ischemic strokes constitute between 60%–70% of all strokes [1, 2]. Ischemic stroke often originates from the rupture of atherosclerotic plaques and the formation of thrombosis, and the occurrence of cerebral ischemia will directly lead to neuronal damage and death in the infarct area, thereby imposing a substantial clinical burden on affected individuals. Current therapeutic strategies for cerebral ischemia are limited to intravenous thrombolysis and endovascular thrombectomy [3, 4]. Nevertheless, reperfusion after transient cerebral ischemia, while necessary to salvage ischemic tissue, can unexpectedly amplify cellular injury and trigger cerebral ischemia–reperfusion (I/R) damage [5, 6]. In these processes, elevated reactive oxygen species (ROS) production accompanied by enhanced neuroinflammatory activation, cerebral microcirculation disorder, and other molecular events involved in stroke pathology play very important roles [6, 7, 8].

Accumulating research has demonstrated that exercise training exerts broad protective effects on brain function [9]. For example, engagement in regular physical activity appears to confer protection against the development of Alzheimer's disease and delay brain aging in older adults [10, 11]. It has been reported that skeletal muscle possesses secretory properties and can synthesize and release multiple myokines in response to contraction, including cathepsin B, FNDC5, irisin, and IGF‐1. These muscle‐derived factors participate in controlling hippocampal functions. Evidence suggests that circulating cathepsin B is capable of entering the brain, where it enhances brain‐derived neurotrophic factor signaling and supports neurogenic processes associated with cognitive function [12], and irisin was reported to reduce brain injury in ischemia and reperfusion mice [13].

Myonectin, a muscle‐derived secretory factor also designated as complement C1q/TNF‐related protein 15 (CTRP15), is encoded by the erythroferrone (*Erfe*) gene and has also been referred to as erythroferrone in previous studies. As the 15th member of the CTRP family, myonectin participates in the modulation of fatty acid metabolism as well as iron homeostasis [14]. As a relatively late‐found myokine, myonectin is mainly expressed in skeletal muscle. Voluntary exercise is able to up–regulate its mRNA and circulating protein levels [15]. Research has shown that myonectin facilitates fatty acid utilization, lowers plasma lipid levels, and enhances systemic insulin responsiveness [16]. Moreover, myonectin alleviates myocardial ischemia–reperfusion injury by limiting apoptotic loss of cardiomyocytes and modulating the inflammatory response of macrophages [17]. However, it remains unclear whether myonectin, like other myokines, participates in muscle–brain communication and confers neuroprotection following cerebral injury. Herein, we investigated the therapeutic effect of exercise‐induced myonectin in cerebral ischemia–reperfusion injury and its underlying mechanism involving neuronal survival and microglial activation.

## 2 Materials and Methods

### 2.1 Mice

The animals consisted of 6–8‐week‐old wild‐type C57BL/6J male mice (Beijing Vital River). *Erfe* ^*flox/flox*^ (hereafter referred to as Flox) mice and skeletal muscle‐specific Myonectin‐deficient mice (*Myf5*‐Cre;*Erfe* ^*flox/flox*^, hereafter referred to as Myo‐mKO) were acquired from Saiye (Suzhou) Biological Technology Co. Ltd. According to the supplier, the Myo‐mKO mouse was established by crossing *Myf5*‐Cre mice with Flox mice, resulting in Cre‐mediated deletion of the Myonectin‐encoding gene *Erfe* in *Myf5*‐expressing skeletal muscle lineage cells. Verified breeding pairs were provided by the vendor, and subsequent colony expansion and maintenance were performed in our animal facility. Flox littermates lacking the Cre transgene served as control animals.

### 2.2 MCAO/R Model

The MCAO/R models were prepared using a wire‐plug technique. Mice were anesthetized with isoflurane (#2024012501, RWD Life Science and Technology, Shenzhen) and positioned supine on a surgical microscope stage under continuous isoflurane inhalation anesthesia. After exposing the cervical vessels, a silicone‐coated nylon filament (#162320, Beijing Xinong Technology Co. Ltd.) was introduced via the external carotid artery and advanced along the internal carotid artery to occlude the MCA at its origin, where it was used to produce transient occlusion. CBF was evaluated using the LSCI system (RFLSI‐ZW, RWD Life Science, China); the exclusion criteria were predefined to exclude animals in which MCAO failed to reduce ipsilateral cortical CBF to 30% ≤ CBF < 40% of baseline values. After maintaining MCA occlusion for 60 min, the intraluminal filament was withdrawn to restore cerebral blood flow and initiate reperfusion. For sham controls, all surgical steps were performed identically except that the filament was not inserted. Rectal temperature was monitored during MCAO surgery and maintained within the physiological range (37.0°C ± 0.5°C) using a feedback‐regulated heating device (Model 69,027, RWD Life Science, China). Animals that exhibited abnormal temperature fluctuations were excluded according to our predefined criteria. Mice which were subjected to exercise preconditioning prior to MCAO/R performed treadmill exercise (KW‐PT, Calvin Biotechnology Co. Ltd., Nanjing, China) at 10 m/min, with each session lasting 90 min daily, starting 30 days before MCAO/R induction. At 24 h after reperfusion, neurological deficits were assessed. Peripheral blood samples, brain tissue, and skeletal muscle samples were then collected for subsequent analyses.

### 2.3 Neurological Function Score

Neurological function was assessed with a 28‐point rodent Neurological Deficit Score scale by evaluators blinded to group information. The assessment included seven neurological domains: postural symmetry, locomotion, climbing performance, circling behavior, forelimb coordination, and whisker reflexes. Each domain was graded from 0 (normal) to 4 (maximal deficit), yielding a cumulative score that was evaluated on a 0–28‐point scale, in which higher scores reflected greater neurological dysfunction. Neurological outcomes were evaluated after a 24‐h reperfusion period following transient MCA occlusion [18].

### 2.4 TTC Staining

After euthanasia, cerebral samples were processed into uniform 2‐mm coronal sections, followed by incubation in 2% TTC staining buffer (#60508ES25, Yeasen Biotechnology (Shanghai) Co. Ltd.) solution at 37°C under light‐protected conditions for 15–30 min, with occasional gentle agitation. Following staining, the coronal sections were imaged, and the infarct area was analyzed using ImageJ.

### 2.5 H&E and Immunohistochemistry Staining

Brain tissues were fixed, paraffin‐embedded, and sectioned. H&E staining and immunohistochemistry were performed using antibodies against Iba‐1 (#RMAB50732, Bioswamp Co. Ltd.), NeuN (#H681904026, HUABIO Biotechnology Co. Ltd.), p‐AKT (#ET1607‐73, HUABIO Biotechnology Co. Ltd.), and p62 (#HA721171, HUABIO Biotechnology Co. Ltd.). After secondary antibody addition and DAB visualization, specimens were dehydrated, mounted, and imaged by microscopy (WS‐10, Zhiyue Medical Technology).

### 2.6 TUNEL Staining

Apoptotic cells were identified using a TUNEL kit(#E‐CK‐A322, Wuhan Elabscience Biotechnology Co. Ltd.). Following deparaffinization and rehydration, tissue sections underwent Proteinase K digestion followed by incubation with the TdT labeling reaction mixture and reaction buffers (37°C, protected from light, 60 min), followed by another PBS wash. DAPI staining was performed for nuclear visualization, followed by mounting with Antifade medium and observed under a microscope (Cytation, BioTek, Winooski, Vermont, USA).

### 2.7 Nissl Staining

Paraffin‐embedded sections underwent dewaxing and graded ethanol rehydration. Nissl dye reagent (#C0117, Beyotime) was then applied, followed by incubation at 37°C for 1 h while being protected from light exposure. Sections were rinsed, dehydrated, resin‐mounted, and examined microscopically (Cytation, BioTek, Winooski, Vermont, USA) for imaging.

### 2.8 Elisa

The myonectin expression of mouse serum and brain was analyzed using the CTRP15 ELISA kit (#F43377‐A, Fancovi). IL‐1β, TNF‐α, and IL‐6 in circulating blood, brain tissue, as well as cell supernatants were detected using Mouse IL‐1β (#1210122) and TNF‐α ELISA Kit (#1217202) from Dakewe Biotech Co. Ltd., Mouse IL‐6 ELISA Kit (#CME0006, Suzhou Sibaozheng Biotechnology Co. Ltd.), respectively. ELISA experiments were conducted following the manufacturer's protocols.

### 2.9 Mouse Genotyping

Genomic DNA was extracted by proteinase K digestion, amplified by PCR with the primers provided below, and analyzed by 1% agarose gel electrophoresis. *Erfe*‐F:5‘‐CATAGAAGCCTCCATCATCCTGA–3’; *Erfe*‐R:5‘‐CAAGAGGCCTTAGAAG A AGTCAAG–3’; *Myf5*‐F:5‘‐ACGAAGTTATTAGGTCCCTCGAC‐3’;*Myf5*‐R:5‘‐CGGCTCTT AAAGCAATGGTC‐3’; Tissue‐specific gene‐F:5‘‐CATAGAAGCCTCCATCATCCTGA‐3’; Tissue‐specific gene‐R:5‘‐TACTGGAAGAAACACAAAGGTTCG‐3’.

The genotyping strategy used a multiplex PCR approach. Based on the primer binding sites and the deleted DNA fragment, the first primer pair amplifies a 135‐bp fragment from *Erfe* ^+/+^ DNA, both 135‐bp and 204‐bp fragments from *Erfe* ^+/−^ DNA, and a single 204‐bp fragment from *Erfe* ^−/−^ DNA. The second primer pair detects the *Myf5*‐Cre allele, yielding an approximately 120‐bp amplicon. No detectable band was observed, suggesting that the *Myf5*‐Cre transgene was not present. An additional third primer pair was included to confirm Cre recombinase activity. A product of approximately 259 bp is generated only in the presence of active Cre enzyme. No amplification indicates the absence of functional Cre activity.

Genotype Identification: Mice exhibiting PCR products of 204 bp, 120 bp, and 259 bp were identified as Myo‐mKO mice. Mice showing only the 204‐bp product from the first primer pair, with no bands from the second or third primer pairs, were identified as Flox mice.

### 2.10 qRT‐ PCR

Gastrocnemius muscle samples were subjected to RNA isolation with TRIzol according to the manufacturer's instructions (#SD1412, Takara, Japan), reverse‐transcribed into cDNA (#AE311, TransGen Biotech, China), and analyzed by SYBR Green qRT‐PCR (#AH0105, Shandong SparkJade Biotechnology Co. Ltd.) with β‐actin as the control gene. Relative changes in *Erfe* mRNA abundance were evaluated through the comparative Ct method. Primers: *Erfe‐F:5′‐*TCTGCACGTGGCACTCACC‐3′, *Erfe*‐R:5′‐AGGTCCAGTGGGCTTCCTTG‐3′.

### 2.11 Recombinant Myonectin Protein Generation

A recombinant plasmid encoding C‐terminal FLAG‐tagged human CTRP15 was constructed by cloning its ORF into pcDNA3.1‐3xFlag‐C (#VT9221, YouBio Technology). The plasmid was transfected into HEK‐293 cells via calcium phosphate. After 48 h, cells were switched to vitamin C‐supplemented serum‐free Opti‐MEM I. Supernatants were collected every 48 h for three cycles and pooled. FLAG‐tagged protein was isolated by affinity purification and released using FLAG peptide, dialyzed in HEPES‐NaCl buffer, quantified, aliquoted, and stored at −80°C.

### 2.12 Intravenous Injection of Myonectin

MCAO/R mice received a single intravenous administration of recombinant myonectin which was dissolved in saline (200 μg/kg body weight, 100 μL saline) or vehicle control (100 μL saline) via the tail vein at 30 min before the induction of ischemia.

### 2.13 Cell Culture

HT22 and BV2 cells were grown in DMEM enriched with 10% FBS and incubated at 37°C in a 5% CO_2_‐controlled environment. OGD/R was induced by glucose/serum deprivation under hypoxia (95% N_2_/5% CO_2_) for 6 h, followed by 22 h of reoxygenation in complete medium. Recombinant mouse myonectin (5 or 10 μg/mL) was added before OGD/R and during reoxygenation. LY294002 (30 μM), 3‐MA (1 mM), or rapamycin (50 nM) was applied as indicated.

### 2.14 Cell Viability

Cells seeded in 96‐well plates were treated with 10 μL CCK‐8 reagent (#C0005, TargetMol), mixed well, incubated for 2 h, followed by the determination of optical density at 450 nm using a microplate spectrophotometer (Epoch2, BioTek, Winooski, Vermont, USA). Calculated the relative viability changes of cells in every group.

### 2.15 Western Blotting

Cells were homogenized in lysis buffer on ice for 30 min and subsequently heated at 95°C for 15 min. The extracted proteins were resolved by SDS‐PAGE and electro‐transferred onto nitrocellulose membranes. Following membrane blocking, incubation with specific primary antibodies was performed at 4°C overnight. The primary antibodies used were obtained from HUABIO Biotechnology (PI3K, #HA722522; p‐PI3K, #HA721672), Cell Signaling Technology (CST) (AKT, #4091; p‐AKT, #4060; mTOR, #2972; p‐mTOR, #2974; ERK1/2, #4695; p‐ERK1/2, #4370; P38 MAPK, #9212; p‐P38 MAPK, #4511; AMPK, #2532; p‐AMPK, #2535; NF‐κB p65, #4764; p‐NF‐κB p65, #3033; P62, #5114; LC3 I/II, #12741; Bcl‐2, #4223; Bax, #5023), EPITOMICS (Beclin‐1, #YK092313DPS), ABclonal (GAPDH, #AC002; β‐tubulin, #AC021), Bioswamp (Iba‐1, #RMAB50732), and Suzhou Kanghengxin Biotechnology Co. Ltd. (CTRP15, #CQA9965). After washing, the membranes were subsequently exposed to HRP‐conjugated secondary antibodies, including goat anti‐mouse IgG (#0291115) and goat anti‐rabbit IgG (#0293120) (Yeasen) for 1 h. Protein bands were visualized by an enhanced ECL detection system (5200Multi, Tanon Technology, Shanghai, China), and the corresponding signal intensities were analyzed using ImageJ software.

### 2.16 Cell Apoptosis

Apoptotic cells were quantified by Annexin V‐FITC/PI staining (#Y6002L, UELandy Biotechnology, Suzhou, China) followed by flow cytometric analysis according to the manufacturer's instructions.

### 2.17 Mitochondrial Membrane Potential

Mitochondrial membrane potential was monitored by JC‐1 dye accumulation using a commercial kit (#E‐CK‐A301, Elabscience). Cells were exposed to JC‐1 solution at 37°C for 20 min and subsequently subjected to washing steps before flow cytometric evaluation.

### 2.18 Mitochondrial Damage

Mitochondrial damage was evaluated using MG and MDR fluorescent probes (#C1048 and #C1032, Beyotime). Cells were stained for 20 min with the probes at 37°C in darkness, rinsed with PBS, followed by flow cytometric evaluation. Low MDR/MG fluorescence indicated mitochondrial impairment.

### 2.19 Reactive Oxygen Species

Intracellular ROS was assessed through a DCFH‐DA‐based ROS detection kit (#S0033S, Beyotime). After a 30‐min incubation with the probe under 37°C conditions, cell samples were washed with PBS and analyzed using flow cytometry.

### 2.20 Monodansylcadaverine Staining

MDC labeling was performed by incubating HT22 cells with 50 μM MDC (#C3019S, Beyotime) for 40 min, followed by PBS washing and confocal imaging. Autophagic fluorescence intensity was quantified.

### 2.21 Statistical Analysis

Statistical analyses were conducted with GraphPad Prism 8.0. Normality and variance consistency were evaluated before testing. Two‐group comparisons used two‐tailed unpaired Student's *t*‐test, while multiple‐group analyses were performed by one‐way ANOVA with Tukey's post hoc test. Nonparametric data were analyzed using Mann–Whitney U or Kruskal–Wallis tests with Dunn's correction. Data are presented as mean ± SD, and *p* < 0.05 was considered significant.

## 3 Results

### 3.1 Endurance Exercise Is Involved in the Cerebral Infarct‐Sparing Effect Related to Myonectin

**FIGURE 1:**
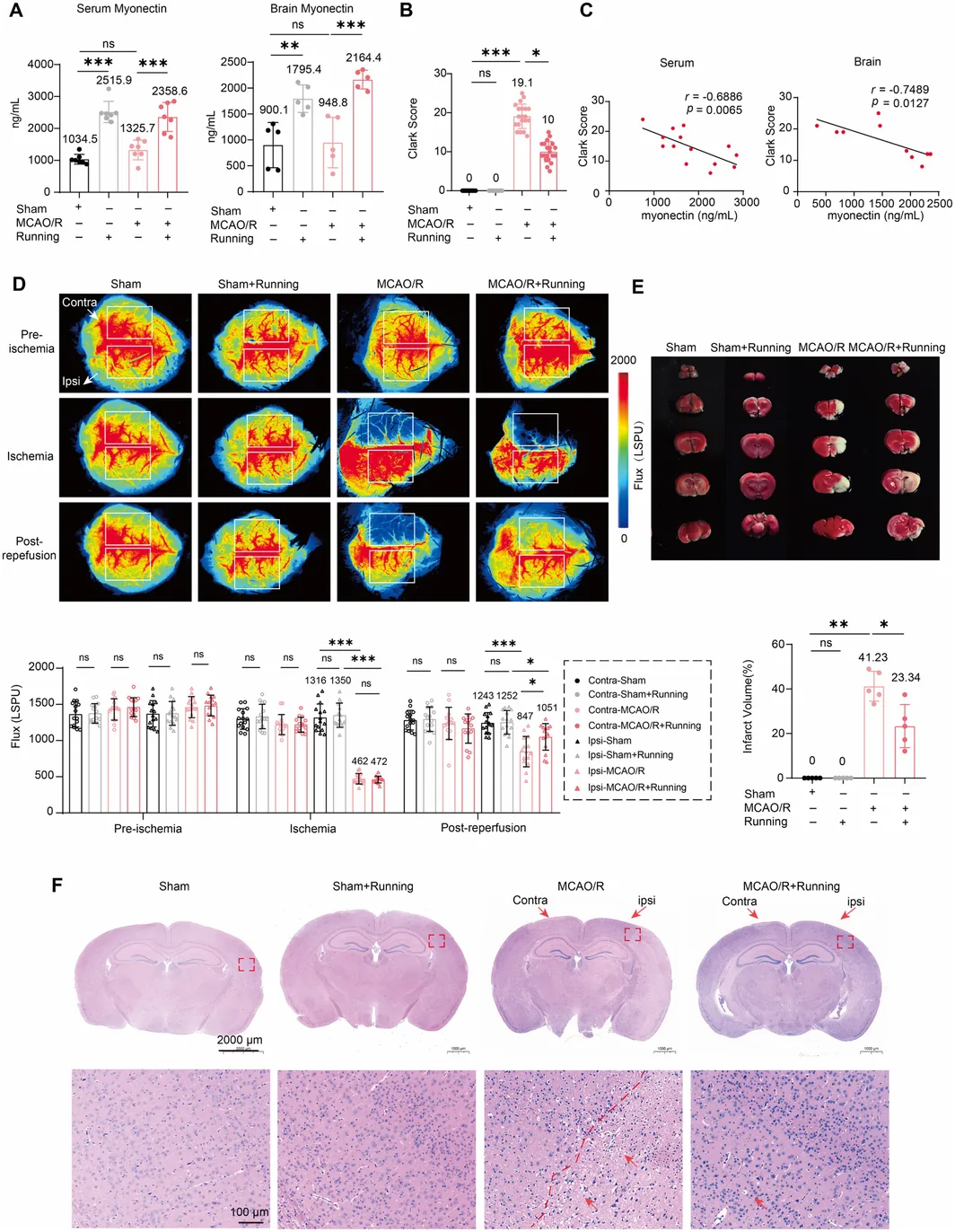
Endurance exercise is involved in the cerebral infarct‐sparing effect related to myonectin. (A) Expression of myonectin in circulating blood and brain from Sham, Sham + Running, MCAO/R, and MCAO/R + Running mice at 24 h after I/R as determined by enzyme‐linked immunosorbent assay (*n* = 7 for blood samples; *n* = 5 for brain samples). (B) Neurological deficit scores (*n* = 20). (C) Correlation between serum and brain myonectin levels and neurological deficit score, *n* = 7 for serum samples, *n* = 5 for brain samples. (D) Representative photos and quantitative analysis of stabilized cerebral blood flux recorded at pre‐ischemia, ischemic, and reperfusion stages in Sham, Sham + Running, MCAO/R, and MCAO/R + Running groups (*n* = 15). LSPU represents Laser Speckle Perfusion Unit; Contra represents contralateral non‐ischemic hemisphere; Ipsi represents ipsilateral ischemic hemisphere. (E) Representative TTC‐stained coronal brain sections and infarct volumes in each group (*n* = 5). (F) Representative coronal brain sections subjected to H&E staining (upper panels) and enlarged images of the ipsilateral cortical regions of interest (lower panels) (*n* = 5). Scale bars: 2000 μm for upper images and 100 μm for lower images. The red arrow indicates the vacuolar degeneration. The red dashed line demarcates the boundary between the infarcted and non‐infarcted regions on the ischemic hemisphere. The left and right panels indicate the contralateral and ipsilateral sides, respectively. The same orientation is used for all immunofluorescence and immunohistochemistry images presented in this study. One‐way ANOVA with Tukey's post hoc test (A, D) or Kruskal‐Wallis with Dunn's multiple comparisons test (B, E) was used. A significant negative correlation was determined by Pearson's correlation analysis (C). Data are presented as the mean ± SD. * *p* < 0.05, **p < 0.01, ***p < 0.001 and ns means no significance.

To investigate whether myonectin contributes to the exercise‐induced protection against cerebral ischemia–reperfusion injury, mice were randomized into four groups: the Sham group, the Sham+Running group, the MCAO/R group, and the MCAO/R + Running group. The MCAO/R model was established by inducing cerebral ischemia for 60 min and subsequently allowing reperfusion for 24 h. Meanwhile, the MCAO/R + Running group ran on a treadmill for 90 min per day for 30 days before being subjected to the same 60 min of cerebral ischemia and 24‐h reperfusion procedure. A comparison of serum and brain myonectin levels was conducted between the Sham and MCAO/R groups. The comparison between the two groups revealed no significant differences, suggesting that MCAO/R surgery did not affect serum and brain myonectin levels. In addition, compared with the Sham group, the Sham + Running mice exhibited significantly upregulated myonectin concentrations in both serum and brain tissues, indicating that myonectin is an exercise‐responsive myokine. Importantly, compared to the MCAO/R group, MCAO/R + Running group markedly elevated circulating myonectin concentrations in serum by 1.78‐fold, and the exercise induced a remarkable 2.28‐fold elevation of myonectin within the brain (Figure 1A). The neurological deficit score is generally used to quantify neurological injury severity [18, 19]. The MCAO/R + Running mice showed significantly lower neurological scores (10 ± 2.596) than the MCAO/R mice (19 ± 3.127), corresponding to a reduction of approximately 47.4% (Figure 1B). Furthermore, a strikingly significant inverse relationship between the neurological deficit score and myonectin levels was revealed in this experiment, in both serum (*r* = −0.6886, *p* < 0.05) and brain (*r* = −0.7489, *p* < 0.05), as illustrated from Figure 1C, indicating that higher expression levels of myonectin in the serum and brain are correlated with less severe neurological functional deficits in mice. To directly visualize cerebral perfusion changes, LSCI was performed at baseline, during MCAO, and after reperfusion. Baseline cerebral blood flow was comparable between the four groups. The marked reduction in ipsilateral cerebral blood flow during MCAO confirmed successful ischemic induction, with similar blood flow reductions detected in the MCAO/R and MCAO/R + Running mice. Notably, the MCAO/R + Running mice exhibited significantly improved cerebral blood flow recovery in the ischemic hemisphere after reperfusion relative to MCAO/R‐operated mice (Figure 1D). Cerebral infarction volume was quantified by TTC staining. TTC analysis revealed a 40.6% reduction in ischemic infarct volume in the MCAO/R + Running group compared with the MCAO/R group (Figure 1E). To consolidate the protective effects of endurance exercise, H&E staining was performed to examine histological alterations in brain tissues. No evident histological abnormalities were detected in the Sham group and Sham+Running groups; nevertheless, MCAO/R mice revealed irregular cellular architecture and marked edema formation. Specifically, the cerebral parenchyma in the infarcted area displayed a reticulated, spongy appearance due to extensive vacuolization and microcavity formation. Exercise preconditioning markedly alleviated histopathological damage in MCAO/R mice (Figure 1F). Collectively, these data provide evidence that endurance exercise‐induced elevation of myonectin is associated with cerebral protection against ischemia–reperfusion injury.

### 3.2 Exercise Exerts a Neuroprotective Effect During Ischemic Reperfusion Injury in Mice

**FIGURE 2:**
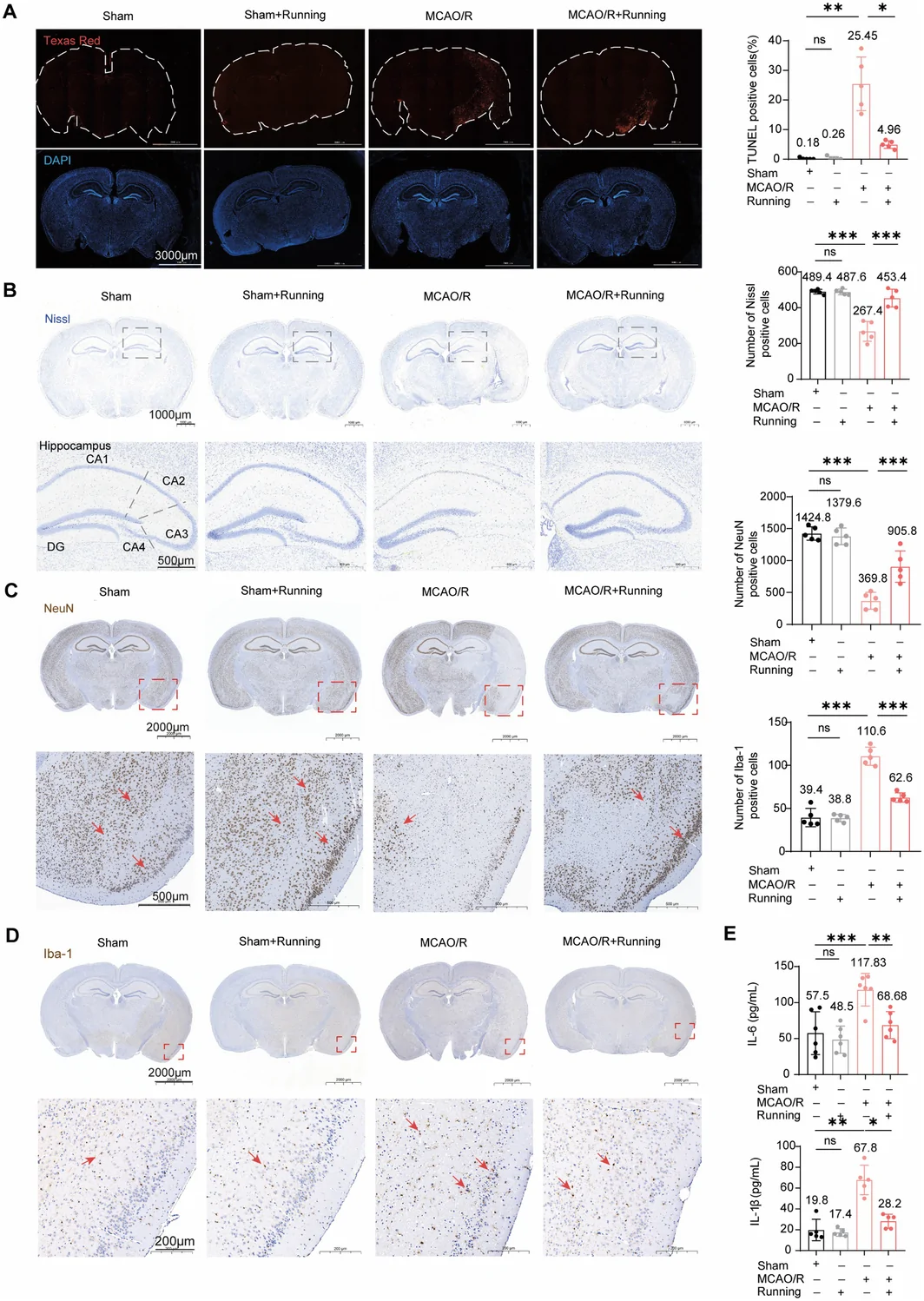
Exercise exerts a neuroprotective effect during cerebral ischemia–reperfusion injury in mice. (A) TUNEL staining and apoptotic cell quantification in brain sections from each group 24 h after sham or I/R treatment. Scale bar: 3000 μm (*n* = 5). TUNEL‐positive nuclei were quantified as a percentage of total nuclei in randomly selected fields. The white dashed lines indicate the boundaries of the coronal brain sections. (B) Nissl‐positive cell staining and quantitative assessment in coronal brain sections and ipsilateral hippocampal subregions, including CA1–CA4 and the dentate gyrus (DG). Scale bars: 1000 μm (top) and 500 μm (bottom); *n* = 5. (C) NeuN‐positive neuron staining images based on immunohistochemistry in coronal brain sections and enlarged images of the ipsilateral cortical regions of interest. Scale bars: 2000 μm (top) and 500 μm (bottom); *n* = 5. (D) Representative Iba‐1 immunohistochemical staining of coronal brain sections (upper panels) and enlarged views of the ipsilateral cortical regions of interest (lower panels). Scale bars: 2000 μm (top) and 200 μm (bottom); *n* = 5. Quantitative analysis of immunopositive (brown) cells (B, C, D). Quantification was performed in randomly selected fields. (E) Serum IL‐6 and IL‐1β concentrations were quantified by ELISA in each group (IL‐6, *n* = 6; IL‐1β, *n* = 5). One‐way ANOVA with Tukey's post hoc test (B–E) or the Kruskal–Wallis test with Dunn's post hoc test (A) was applied. Data are presented as the mean ± SD. * *p* < 0.05, **p < 0.01, ***p < 0.001 and ns means no significance.

A range of pathological alterations, including neuronal apoptosis as well as inflammatory activation, contribute to ischemia–reperfusion injury, leading to further neuronal damage at cellular and tissue levels [20]. To explore the neuroprotective effects of endurance exercise, TUNEL staining was first performed to assess cellular apoptosis in brain tissue 24 h post‐reperfusion. The Sham and Sham+Running mice exhibited negligible numbers of apoptotic cells. Relative to sham mice, MCAO/R markedly increased the abundance of apoptotic cells within the ischemic brain regions. Notably, the MCAO/R + Running group showed a marked attenuation of MCAO/R‐induced apoptotic cell accumulation, as evidenced by a 5.13‐fold decrease in TUNEL‐positive cells compared with the MCAO/R group (Figure 2A). Nissl bodies are distinctive features within neuronal cell bodies [21]. Nissl staining revealed a pronounced reduction in hippocampal Nissl body abundance in MCAO/R mice compared with sham controls, especially in hippocampal CA1‐CA4 subfields. The loss of Nissl bodies induced by MCAO/R was substantially attenuated in the MCAO/R + Running group, resulting in a 69.6% increase in Nissl body numbers (Figure 2B). NeuN has been extensively validated as a marker of mature neurons, with an increase in NeuN‐positive cells generally indicating improved neuronal survival [22]. MCAO/R‐induced neuronal loss was alleviated in the MCAO/R + Running group, resulting in a marked elevation of NeuN‐positive cell abundance (Figure 2C). Central nervous system‐resident microglia rapidly respond to ischemia–reperfusion stress by transitioning into an activated state. Iba‐1 is frequently utilized as a reliable marker for identifying activated microglia [23]. The MCAO/R group exhibited greater Iba‐1 immunoreactivity than the Sham mice, indicating significant microglial activation. Conversely, exercise preconditioning mitigated I/R‐induced microglial reactivity, accompanied by a 43.4% decrease in Iba‐1‐positive cells (Figure 2D). Following activation, microglia contribute to neuroinflammation by releasing cytokines including IL‐6 and IL‐1β, which are critically involved in neuroinflammation [24]. Consistent with this, following exercise intervention, the elevations of IL‐6 and IL‐1β induced by MCAO/R were significantly attenuated, showing reductions of 41.7% and 58.4%, respectively (Figure 2E). Together, our findings reveal that exercise provides neuroprotection against ischemia–reperfusion damage through suppressing neuronal apoptosis as well as alleviating neuroinflammation.

### 3.3 Muscle‐Derived Myonectin Alleviates Cerebral Ischemia–Reperfusion Injury

**FIGURE 3:**
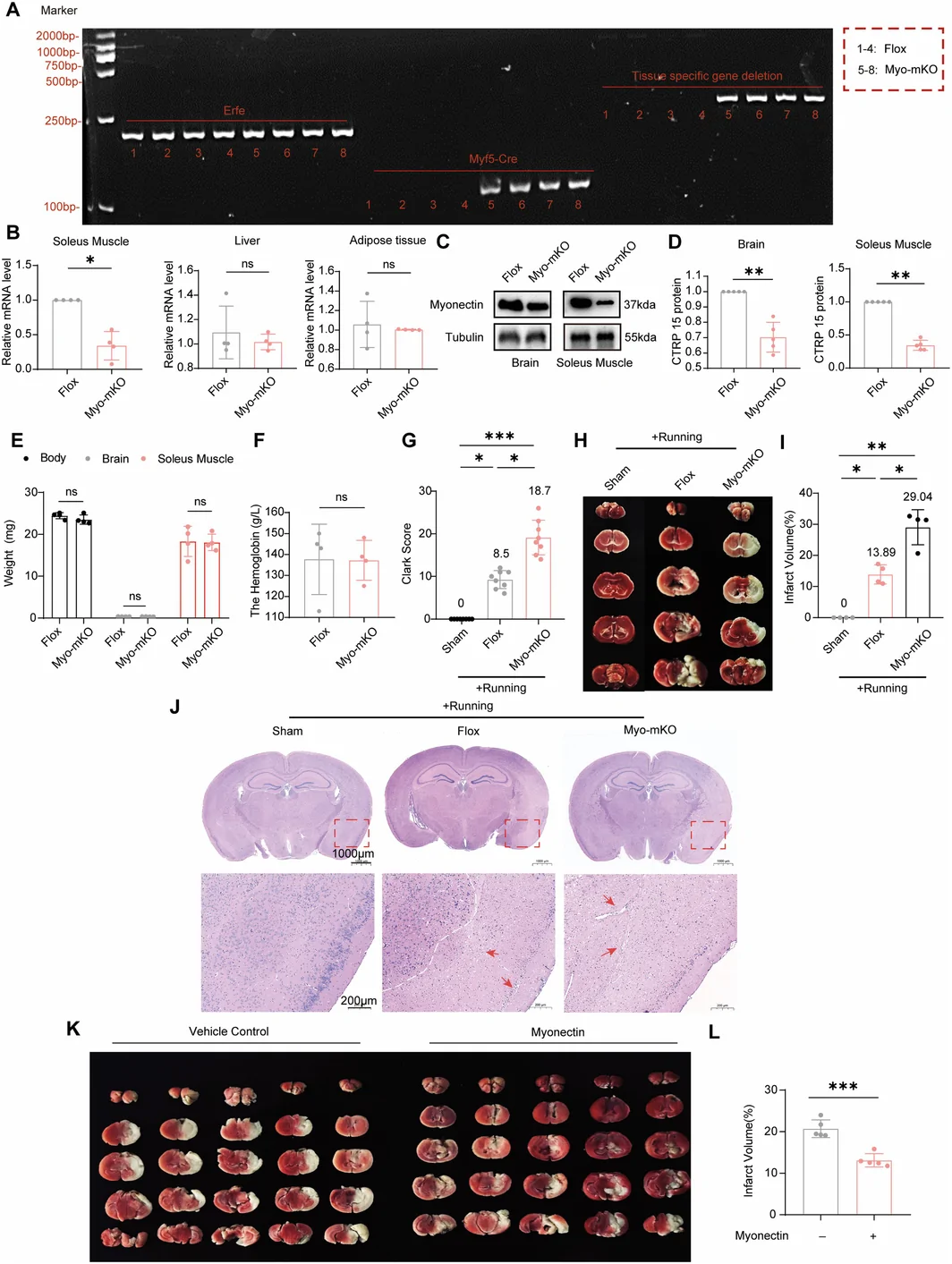
Muscle‐derived myonectin alleviates cerebral I/R injury. (A) Detection of PCR‐amplified myonectin genomic DNA fragments in Flox and Myo‐mKO mice by agarose gel electrophoresis with three primer sets (*n* = 8). (B) Myonectin mRNA levels in the soleus muscle, liver, and adipose tissue were measured by qRT‐PCR in Flox and Myo‐mKO mice (*n* = 4). (C) Representative immunoblots and (D) Relative myonectin protein abundance in the brain and soleus of Flox and Myo‐mKO mice determined by western blot (*n* = 5). (E) Body, brain, and skeletal muscle weights in sham, Flox and Myo‐mKO groups (*n* = 4). (F) Hemoglobin concentration in sham, Flox and Myo‐mKO groups (*n* = 4). (G) Neurological deficit scores in sham, Flox and Myo‐mKO groups after endurance exercise (*n* = 8). (H) Representative TTC‐stained brain sections. (I) Quantification of infarct volume in sham, Flox and Myo‐mKO groups after endurance exercise (*n* = 4). (J) Representative H&E‐stained sections of the cerebral cortex and enlarged images of the ipsilateral cortical regions of interest in sham, Flox and Myo‐mKO groups after endurance exercise (*n* = 3). Scale bars: 1000 μm (overview images) and 200 μm (enlarged images). (K) Representative TTC staining of brain sections. (L) Relative infarct volume in vehicle‐ and myonectin‐treated mice (*n* = 5). Group comparisons were performed using the Mann–Whitney U test (B, soleus and liver; D), unpaired two‐tailed Student's *t*‐test (B, adipose tissue; E, F, L), or Kruskal–Wallis/Dunn's post hoc test (G, I). Data are presented as the mean ± SD. **p < 0.01, ***p < 0.001.

To validate the successful generation of skeletal muscle‐specific Myonectin‐deficient mice, genotyping analysis was performed and confirmed the expected Myf5‐Cre‐mediated recombination in Myo‐mKO mice, whereas the unrecombined floxed allele was retained in Flox control mice (Figure 3A). To confirm the tissue specificity of myonectin deletion, myonectin mRNA levels were examined in the soleus muscle, liver, and adipose tissue of Myo‐mKO and Flox mice. Compared with Flox mice, Myo‐mKO mice showed markedly decreased myonectin mRNA levels in the soleus muscle, whereas its expression in the liver and adipose tissue remained unchanged (Figure 3B), suggesting that skeletal muscle is the primary affected tissue following Myf5‐Cre‐mediated myonectin deletion. Western blot analysis demonstrated a marked reduction in myonectin levels in Myo‐mKO mice, with protein expression in the brain and soleus muscle being significantly decreased relative to Flox mice (Figure 3C,D). No alterations in body weight or brain weight, and skeletal muscle weight between Myo‐mKO mice and Flox mice were detected (Figure 3E). Moreover, there were no disparities in hemoglobin levels between the two mouse strains (Flox mice: 137.75 ± 16.76 g/L; Myo‐mKO mice: 137.25 ± 9.46 g/L) (Figure 3F). Thus, under basal physiological conditions, Myo‐mKO mice were indistinguishable from Flox mice.

In comparison to Flox mice, Myo‐mKO mice exhibited a significantly elevated neurological deficit score (increased by 2.2‐fold) after cerebral ischemia–reperfusion following endurance exercise (Figure 3G). Concomitantly, the cerebral infarction volume increased markedly by 52.2% (Figure 3H,I). Moreover, H&E staining of the cerebral cortex was used to assess the degree of cortical damage. Compared with Flox mice, Myo‐mKO mice presented more severe tissue edema and neuronal loss in the cerebral cortex; however, no such injuries were detected in the brains of the sham group (Figure 3J). These data support the contribution of muscle‐derived myonectin in mediating the beneficial effects of endurance exercise on ischemia–reperfusion injury.

Furthermore, to evaluate the protective effect of myonectin administered prior to cerebral ischemia–reperfusion–induced brain injury, a human recombinant myonectin protein was produced for subsequent experiments. Wild‐type C57BL/6J mice received a single intravenous injection of recombinant myonectin protein or vehicle control (an equal volume of saline) 30 min prior to 60 min of ischemia induction followed by 24 h of reperfusion. Myonectin treatment significantly diminished infarct volume after I/R injury relative to the vehicle control (Figure 3K,L). The results indicate that systemic myonectin protein administration exerts a protective effect against I/R injury and may represent a promising therapeutic approach.

### 3.4 Muscle‐Derived Myonectin Suppresses Neuronal Apoptosis and Microglial Inflammatory Responses

**FIGURE 4:**
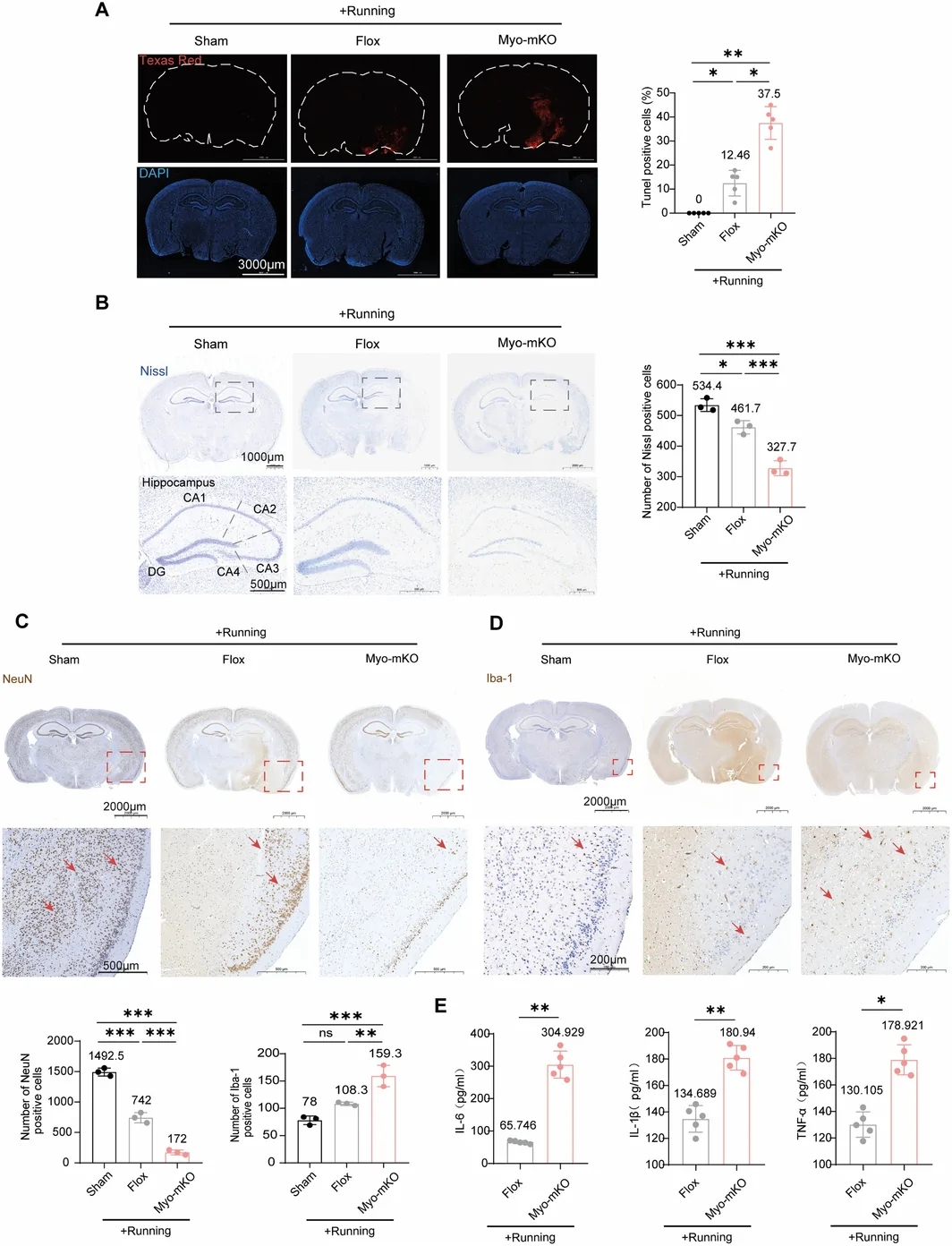
Muscle‐derived myonectin suppresses neuronal apoptosis and microglial inflammatory responses. (A) Representative brain sections showing TUNEL staining in Sham, Flox, and Myo‐mKO mice after endurance exercise and ischemia/reperfusion (left). Scale bar: 3000 μm. Percentage of TUNEL‐positive nuclei among total nuclei (right; *n* = 5). (B) Representative Nissl staining images (left) and cell quantification (right) in coronal brain sections and enlarged images of the ipsilateral hippocampus (including CA1–CA4 and DG) by Nissl staining. Scale bars: 1000 μm and 500 μm (*n* = 3). (C) Representative NeuN staining pictures (left) and quantification of NeuN‐positive neurons (right), with enlarged views of the ipsilateral peri‐infarct cortical ROIs. Scale bars: 2000 μm and 500 μm (*n* = 3). (D) Representative Iba‐1 immunostaining of coronal brain sections (left) and quantification of Iba‐1–positive microglia within the ipsilateral cortex (right) ROIs. Scale bars: 2000 μm and 200 μm (*n* = 3). (E) Serum IL‐6, IL‐1β, and TNF‐α levels in Sham, Flox, and Myo‐mKO mice after exercise and I/R were measured by ELISA (*n* = 5). Group comparisons were performed by one‐way ANOVA followed by Tukey's post hoc test (B–E) or the Kruskal–Wallis test followed by Dunn's post hoc test (A). Data are presented as mean ± SD. ns, not significant; * *p* < 0.05, **p < 0.01, ***p < 0.001.

To determine whether myonectin derived from skeletal muscle regulates neuronal apoptosis and microglial inflammatory responses during cerebral I/R, we performed TUNEL staining to assess apoptosis in the ischemic brain tissue 24 h after MCAO/R in mice with muscle‐specific knockout of myonectin. Loss of myonectin abolished the neuroprotective effects of endurance exercise, as evidenced by the increased accumulation of apoptotic cells in the Myo‐mKO group relative to the Flox group (Figure 4A). Nissl staining further revealed a substantial loss of Nissl bodies across the hippocampal CA1–CA4 and DG subregions in Myo‐mKO mice (Figure 4B). In the MCAO/R model, where the lateral cerebral cortex is the core ischemic region [25], NeuN staining demonstrated that Myo‐mKO mice had significantly fewer surviving neurons, indicated by brown NeuN‐positive signals, in the cerebral cortex compared with Flox controls (Figure 4C). Furthermore, myonectin deficiency enhanced microglial activation, as evidenced by increased Iba‐1 protein expression and a 47% increase in Iba‐1‐positive cells in the brain (Figure 4D). Meanwhile, Myo‐mKO mice showed enhanced microglia‐driven inflammatory responses, as indicated by increased production of IL‐6, IL‐1β, and TNF‐α, relative to Flox controls (Figure 4E). These findings suggest that in Myo‐mKO mice, endurance exercise fails to provide neuroprotective benefits, indicating that muscle‐derived myonectin plays an indispensable role in inhibiting neuronal apoptosis and microglia‐driven neuroinflammation after cerebral I/R.

### 3.5 Myonectin Mitigates Autophagy‐Induced Neuronal Cells Apoptosis and Microglial Activation

**FIGURE 5:**
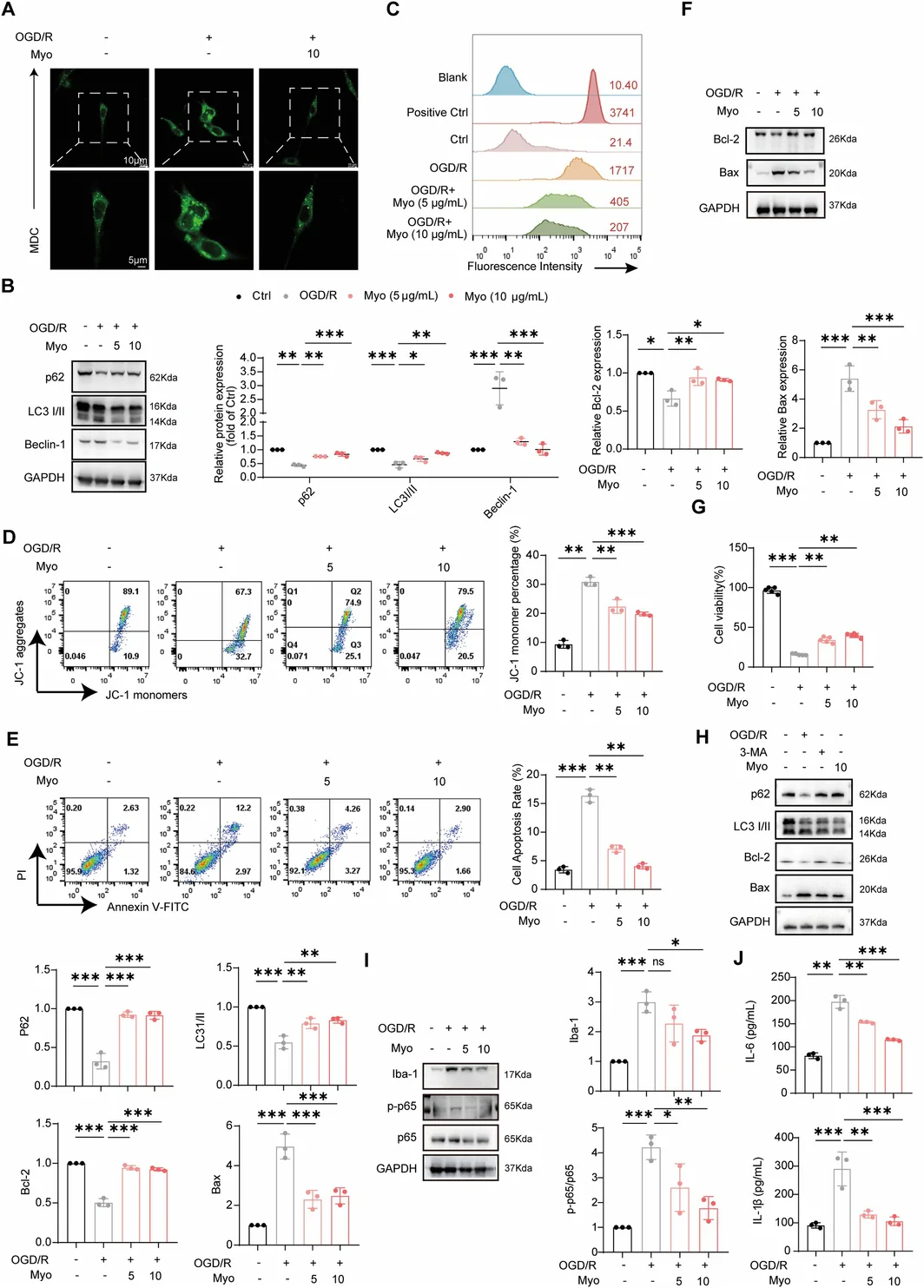
Myonectin attenuates neuronal autophagy and apoptosis and inhibits microglial activation. (A) Autophagosome formation in HT22 cells detected by MDC staining in the control, OGD/R, and 10 μg/mL myonectin (Myo)‐treated groups, Scale bars: 10 μm (top), 5 μm (bottom). (B) Immunoblotting assessment of p62, Beclin‐1, and LC3‐I/II protein expression in HT22 cells. (C) DCFH‐DA staining was used to assess intracellular ROS production in HT22 cells across the indicated groups. (D) JC‐1‐based fluorescence analysis was performed to evaluate mitochondrial membrane potential in HT22 neuronal cell; bar graph shows the percentage of JC‐1 monomers. (E) Quantification of apoptotic HT22 cells by flow cytometry.; bar graphs compare apoptosis ratios. (F) Representative immunoblots and quantification of Bcl‐2/Bax ratios in HT22 cells. (G) Evaluation of HT22 cell viability using the CCK‐8 assay. (H) Protein levels of p62, LC3‐I/II, Bcl‐2, and Bax in HT22 cells determined by immunoblotting.; bar graphs show expression ratios. (I) Western blot detection of Iba‐1 and p‐p65/p65 proteins in BV2 cells, with corresponding densitometric quantification. (J) ELISA‐based quantification of IL‐6 and IL‐1β. One‐way ANOVA with Tukey's post hoc test was applied for statistical analysis. Data represent the mean ± SD from three independent experiments. ns, not significant; * *p* < 0.05, **p < 0.01, ***p < 0.001.

To determine whether myonectin suppresses neuronal apoptosis and microglial activation, we first performed OGD/R on mouse neuronal HT22 cells with or without myonectin. Autophagy is typically induced during the early stage of oxygen–glucose deprivation as an adaptive response that enables cell survival under stress. However, upon recovery, the restoration of oxygen and nutrients often induces excessive autophagy, largely due to increased oxidative stress, ultimately leading to apoptosis. Therefore, strategies that modulate aberrant autophagy‐induced apoptosis during recovery are pivotal for neuronal protection [26]. Exercise‐generated ROS serve as signaling molecules that stimulate the release of myokines, including BDNF, FGF21, IL‐6, IL‐15, and irisin, which systemically regulate redox balance, often suppressing ROS or inducing adaptive antioxidant responses, and establish a beneficial exercise‐adaptation loop [27, 28, 29, 30, 31]. To examine whether myonectin abrogates excessive autophagy in neuronal cells during OGD/R, we first used the fluorescent probe monodansylcadaverine (MDC) to measure autophagosomes. OGD/R markedly increased the number of autophagosomes (green fluorescent puncta), whereas treatment with 10 μg/mL myonectin (abbreviated Myo) dramatically reduced their formation (Figure 5A). During autophagy, Beclin‐1 expression is upregulated to promote the process, and LC3‐I is converted into LC3‐II, which accumulates on the membranes of autophagosomes. Additionally, the autophagic substrate p62 is degraded upon autophagosome‐lysosome fusion [32]. We next assessed autophagy‐associated markers, including p62, LC3‐I/II, and Beclin‐1. Myonectin significantly counteracted OGD/R‐induced alterations in autophagic markers, restoring p62 expression, reducing Beclin‐1 elevation, and suppressing LC3‐I/LC3‐II conversion (Figure 5B), indicating that myonectin inhibits autophagy during OGD/R. Excessive autophagy‐associated oxidative stress leads to ROS accumulation, which damages cellular components [28]. ROS accumulation was markedly enhanced following OGD/R exposure in HT22 cells, resulting in an 81.23% elevation in mean fluorescence intensity (MFI) relative to untreated controls. Myonectin attenuated this increase, reducing ROS levels by 76.4% at 5 μg/mL and by 84.4% at 10 μg mL (Figure 5C). In ischemia–reperfusion injury, the abrupt restoration of oxygen after hypoxia causes a rapid decrease in mitochondrial membrane potential (ΔΨm) [29]. The dye JC‐1, which shifts from red aggregates to green monomers as ΔΨm declines, is commonly used to assess ΔΨm [30]. The proportion of monomers was substantially diminished to 11.04% and 10.25% following exposure to 5 μg/mL and 10 μg/mL myonectin, respectively (Figure 5D). We next detected whether myonectin attenuates apoptosis and improves neuronal survival after OGD/R. Annexin V/PI staining revealed that OGD/R exposure markedly increased apoptotic cell death, whereas myonectin treatment significantly mitigated this effect, with near‐complete inhibition at the higher dose (Figure 5E). Similarly, western blot analysis showed that myonectin concentration‐dependently attenuated apoptosis by decreasing the abundance of Bax while restoring the expression of the anti‐apoptotic protein Bcl‐2 (Figure 5F). Consistent with these results, cell viability was dramatically decreased by OGD/R but was greatly enhanced by 5 μg/mL and 10 μg/mL myonectin, which increased viability 2.15‐fold and 2.51‐fold, respectively, relative to the OGD/R group (Figure 5G). To determine whether neuronal apoptosis is driven by excessive autophagy, the autophagic process was pharmacologically inhibited using 3‐methyladenine (3‐MA). Western blotting showed that both 1 mM 3‐MA and 10 μg/mL myonectin significantly upregulated p62 expression, suppressed LC3‐I to LC3‐II conversion, increased Bcl‐2, and suppressed Bax expression in comparison with the OGD/R group (Figure 5H), indicating that myonectin inhibits OGD/R‐induced neuronal apoptosis by suppressing aberrant autophagy. Collectively, these findings demonstrate that myonectin alleviates ROS‐burst‐triggered excessive autophagy, thereby reducing neuronal apoptosis under hypoxic‐glycolytic stress.

Microglia in the central nervous system can adopt pro‐inflammatory or neuroprotective phenotypes determined by their activation state. Pathogen‐ or damage‐associated pro‐inflammatory cytokines activate resting microglia to produce factors such as IL‐1β, IL‐6, and TNF‐α, which are detrimental to neurons [33]. We therefore evaluated the effect of myonectin on inflammatory activation in BV2 cells exposed to OGD/R. OGD/R markedly upregulated Iba‐1 expression compared with the control, and myonectin (5 and 10 μg/mL) concentration‐dependently attenuated this increase. Phosphorylation of NF‐κB/p65 is a canonical event in inflammatory signaling [31], myonectin also inhibited OGD/R‐induced NF‐κB/p65 phosphorylation in a concentration‐dependent manner (Figure 5I). Furthermore, ELISA showed that myonectin concentration‐dependently reduced IL‐6 and IL‐1β levels, with decreases of approximately 42% and 64%, respectively (Figure 5J). These results suggest that myonectin effectively suppresses inflammatory activation in microglia. In summary, myonectin inhibits autophagy‐induced apoptosis in neuronal cells and inflammatory activation in microglial cells.

### 3.6 Myonectin Provides Neuroprotection via PI3K/AKT/mTOR Signaling Pathway

**FIGURE 6:**
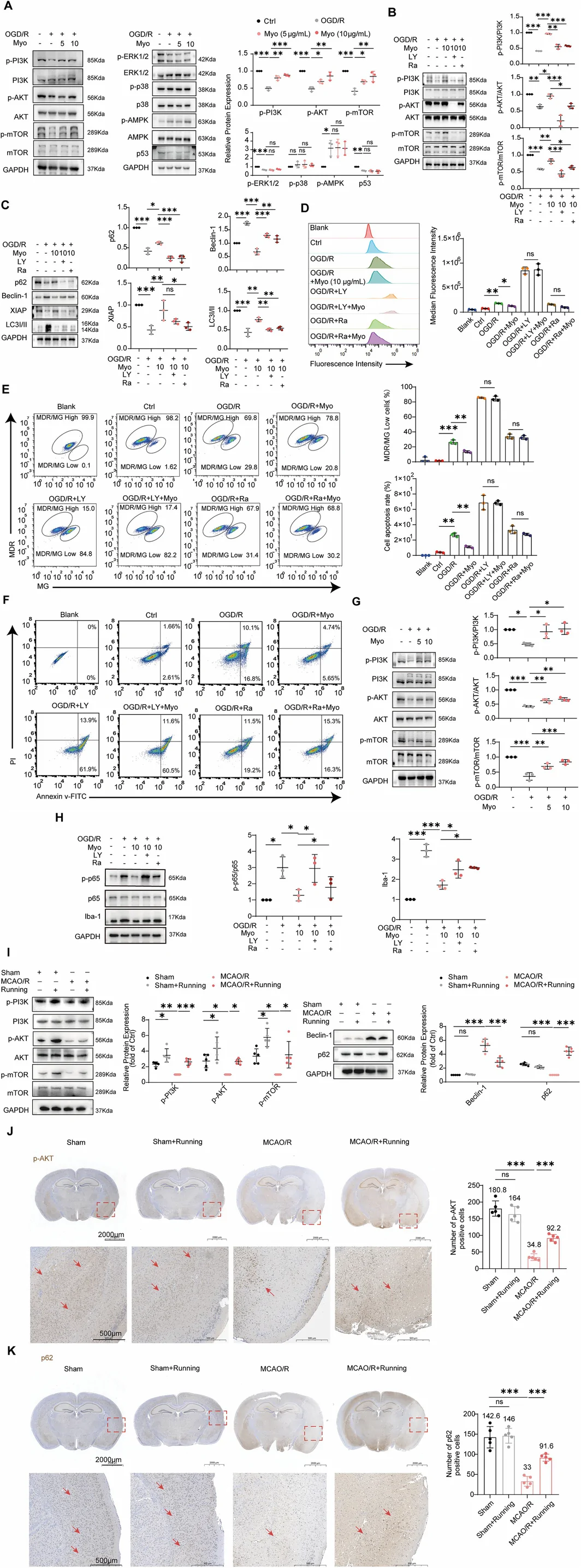
Myonectin suppresses neuronal autophagy, apoptosis, and microglial activation via the PI3K/AKT/mTOR pathway. (A) Western blot detection of PI3K, AKT, mTOR, ERK1/2, p38, and AMPK phosphorylation, together with p53 expression, in HT22 cells. Relative protein ratios were quantified by densitometry. (B) Protein phosphorylation of PI3K, AKT, and mTOR in HT22 cells treated as indicated was evaluated by western blot, presented in bar graphs. (C) Relative protein levels of p62, Beclin‐1, XIAP, and LC3‐I/II in HT22 cells from the same experimental groups as panel B. (D) ROS levels measured by DCFH‐DA staining in HT22 cells across experimental groups, with relative values displayed in bar graphs. (E) Mitochondrial membrane potential (MMP) assessed by MDR/MG staining in HT22 cells, with MDR/MG Low ratios compared in bar graphs. (F) Apoptosis rates in HT22 cells detected by flow cytometry, shown as bar graphs. (G) Immunoblotting analysis of PI3K, AKT, and mTOR phosphorylation in BV2 cells. under different treatments, presented with line graphs. (H) The p‐p65/p65 and Iba‐1 protein level in BV2 cells under indicated conditions, with line graphs comparing ratios. (I) Protein phosphorylation of PI3K, AKT, and mTOR, along with Beclin‐1 and p62 protein expression, was determined by western blot in brain tissues from the four experimental groups. Representative images of (J) p‐AKT‐ and (K) p62‐ positive cells in coronal brain sections and magnified image of the ipsilateral cortical regions of interest (*n* = 3). Scale bars: 2000 μm (top), 500 μm (bottom). Data represent the mean ± SD from three independent experiments. Group comparisons were conducted using one‐way ANOVA with Tukey's post hoc test (A–D, F–K) or the Kruskal–Wallis test with Dunn's post hoc test (E). * *p* < 0.05, **p < 0.01, ***p < 0.001; ns indicates no significance.

To elucidate the underlying mechanism through which myonectin inhibits autophagy in neuronal cells, we examined key signaling molecules that regulate neuronal autophagy. Pathways that activate mTOR, including the Akt, MAPK, and p53 signaling cascades, are known to suppress autophagy, whereas pathways that inhibit mTOR, such as AMPK signaling pathways, promote autophagy [34, 35]. Immunoblotting was performed to assess whether myonectin modulates the expression of four key signaling molecules. The phosphorylation of PI3K, AKT, and mTOR was notably suppressed by OGD/R, while myonectin effectively alleviated these reductions (Figure 6A). It is well established that OGD/R activates autophagy, upregulating p‐AMPK and downregulating p‐ERK1/2 and p53, while changes in p‐p38 remain inconclusive [34, 35]. Consistent with our observations, the phosphorylation levels of AMPK were markedly increased, while those of p‐ERK1/2 and p53 were reduced after OGD/R. We found no change in the protein levels of p‐p38. However, myonectin showed no significant effect on the aforementioned proteins (Figure 6A). Collectively, our data indicate that the PI3K/AKT/mTOR pathway—rather than the ERK1/2, AMPK, or p53 pathways—serves as the dominant route for myonectin‐mediated suppression of autophagy and apoptosis. To confirm this finding, we applied the PI3K inhibitor LY294002 (LY) and the mTOR inhibitor rapamycin (Ra) to determine whether myonectin suppresses autophagic activity in HT22 cells. The OGD/R‐induced enhancement of PI3K/AKT/mTOR signaling by myonectin was markedly attenuated following treatment with LY294002 or rapamycin (Figure 6B). As expected, the alterations in autophagy flux markers—namely enhanced p62 expression together with suppressed Beclin‐1 levels and attenuation of the LC3‐II/LC3‐I ratio detected following myonectin post‐OGD/R—were reversed by LY294002 or rapamycin. Similar opposing effects were seen for the anti‐apoptotic protein XIAP; its elevation in response to myonectin treatment was clearly abolished by both inhibitors in comparison with the OGD/R group (Figure 6C). Furthermore, treatment with LY294002 or rapamycin markedly aggravated OGD/R‐induced oxidative stress, leading to increased ROS levels, which overturned the inhibitory effect of myonectin on ROS generation after OGD/R (Figure 6D). MDR and MG probes were used to assess mitochondrial integrity by detecting membrane potential‐dependent and total mitochondria, respectively. A decrease in the MDR/MG ratio reflects loss of mitochondrial membrane potential, suggesting mitochondrial damage [36]. We observed that PI3K or mTOR inhibitors similarly abolished the ability of myonectin to counteract OGD/R‐induced mitochondrial damage (Figure 6E). Furthermore, the reduction in apoptosis rates mediated by myonectin was also reversed by both inhibitors (Figure 6F). These data suggest that PI3K/AKT/mTOR pathway activation is indispensable for myonectin‐induced inhibition of neuronal autophagy and apoptosis. Given that sustained inflammatory responses and oxidative stress during OGD/R generate excessive ROS, which can subsequently inhibit the PI3K/AKT/mTOR pathway, we hypothesize that the suppressive effects of myonectin on BV2 microglial activation are also mediated through this signaling axis [37, 38]. As anticipated, OGD/R‐challenged BV2 cells exhibited reduced PI3K/AKT/mTOR pathway activation, as evidenced by lower phosphorylation levels; this reduction was significantly reversed by myonectin (Figure 6G). In contrast, compared with the myonectin‐treated group, both inhibitors largely abolished the inhibitory effect of myonectin on OGD/R‐induced Iba‐1 and p‐p65 upregulation (Figure 6H). These findings reveal that myonectin exerts its anti‐inflammatory effects in BV2 cells primarily through PI3K/AKT/mTOR signaling activation. The involvement of myonectin in modulating PI3K/AKT/mTOR signaling and autophagy was further evaluated in vivo by detecting relevant proteins in brain tissues. Immunoblotting revealed enhanced phosphorylation of PI3K, AKT, and mTOR following exercise intervention, accompanied by decreased Beclin‐1 expression and elevated p62 levels relative to MCAO/R mice (Figure 6I). In line with these findings, immunohistochemical staining of coronal brain sections revealed that MCAO/R markedly decreased p‐AKT and p62 levels, whereas exercise restored their expression (Figure 6J,K). Taken together, our findings demonstrate that myonectin exerts neuroprotective effects through promoting PI3K/AKT/mTOR pathway activity and attenuates aberrant autophagic activation across in vivo and in vitro models.

## 4 Discussion

Hundreds of myokines are secreted from muscle cells and have been shown to influence various tissues and organs, specifically the central nervous system. A key challenge is to clarify the link between neuroprotection and myokines. Here, we demonstrate for the first time that myonectin, a kind of myokine, exhibits the potential to mitigate cerebral ischemia–reperfusion damage.

Using multiple approaches, we observed that endurance exercise reduced infarct size and provided neuroprotective effects after cerebral ischemia–reperfusion in the normal mice. However, these beneficial effects were not found in the myonectin‐deficient mice. Furthermore, myonectin was strikingly up‐regulated in the brain and serum, and a significant inverse correlation was found between cerebral blood flow and myonectin levels after physical exercise. In addition, exercise‐induced myonectin exerts neuroprotection after cerebral ischemia by suppressing excessive autophagic neuronal apoptosis and dampening microglia‐driven inflammatory responses. In summary, our data identify myonectin as a critical mediator of exercise‐induced brain protection.

Myonectin has been reported to protect the heart from ischemia–reperfusion injury. Exposure of cultured cardiomyocytes to myonectin protein reduced apoptosis caused by hypoxia/reoxygenation through S1P (sphingosine‐1‐phosphate)‐dependent stimulation of the cAMP/Akt signaling cascade. In parallel, myonectin was found to inhibit the inflammatory reaction triggered by lipopolysaccharide in macrophages via the same pathway [17]. Our research demonstrates that the PI3K/AKT/mTOR pathway participates in the attenuation of neuronal cell apoptosis and microglial cell activation after myonectin treatment. The observed discrepancies may be attributed to variations in cellular context and experimental models. In our study, cells were subjected to oxygen–glucose deprivation/recovery conditions, rather than hypoxia/reoxygenation treatment.

PI3K/AKT activation has been shown to enhance mTOR signaling while suppressing Beclin‐1 expression and limiting LC3‐I/LC3‐II transition, contributing to autophagy suppression following cerebral ischemia–reperfusion [39, 40]. Ischemic stroke is increasingly recognized as a thrombo‐inflammatory disorder [41]. Activation of PI3K/AKT signaling attenuates neuroinflammation after cerebral I/R by limiting inflammatory activation and cytokine production [42]. Moreover, PI3K/AKT signaling mitigates oxidative stress by enhancing antioxidant defenses (Nrf2, SOD, GSH, and GPx) and reducing MDA accumulation, thereby protecting neurons against cerebral I/R injury [43]. In our research, it was observed that myonectin significantly increased the phosphorylation levels of PI3K, AKT, and mTOR. Furthermore, the administration of PI3K and mTOR inhibitors was shown to counteract the myonectin‐induced reductions in autophagy, apoptosis, and ROS production in neuronal cells, as well as the activation of microglial cells. These findings support the conclusion that myonectin provides I/R protection through the PI3K/AKT/mTOR signaling pathway.

The peripheral mechanisms through which exercise elicits these positive effects are not fully understood. Our findings suggest that the increased brain myonectin levels observed after exercise are unlikely due to leakage caused by I/R‐induced BBB disruption. This may be explained by the fact that exercise training alone was sufficient to enhance myonectin levels in the serum and brain tissue of Sham mice in our study. Myonectin is a member of the adiponectin paralog family, also known as the CTRP family. Adiponectin, an adipocyte‐derived protein, can enter the brain after exercise‐induced peripheral elevation by crossing the BBB, thereby promoting neurogenesis and exerting antidepressant effects in neurological conditions, encompassing ischemic stroke, Alzheimer's disease, and multiple sclerosis [44, 45]. We propose that myonectin may traverse the BBB through pathways analogous to those described for adiponectin transport. However, this hypothesis requires further experimental validation. Future studies are warranted to identify the potential transport pathways involved in myonectin entry into the brain.

Adiponectin exerts its metabolic effects primarily through the AdipoR1 and AdipoR2 receptors, which activate distinct signaling pathways: AdipoR1 activates AMPK to regulate energy homeostasis, whereas AdipoR2 is associated with energy utilization and oxidative stress reduction [46]. Whether myonectin, which shares structural homology with adiponectin, utilizes the same receptor(s) as adiponectin remains unclear. Therefore, further studies involving receptor identification, cell‐type‐specific localization, and conditional genetic approaches will be required to determine the predominant cellular targets and signaling mechanisms of myonectin in the brain. In our study, myonectin concurrently activates the PI3K/Akt/mTOR pathway in neurons and microglia, thereby inhibiting excessive neuronal autophagy and attenuating microglial inflammatory responses. However, it remains unclear which cell type is the primary target of myonectin in vivo; a rigorously validated receptor‐blocking tool is indispensable for elucidating its mechanism of action in the future.

In summary, our study identifies myonectin as a new myokine involved in exercise‐induced muscle–brain communication with a previously unrecognized mechanism whereby myonectin activates the PI3K/Akt/mTOR signaling pathway to simultaneously alleviate autophagy‐dependent neuronal apoptosis and attenuate microglial inflammatory activation, thereby providing protection against ischemic brain injury. Importantly, we demonstrate for the first time that exogenous administration of recombinant myonectin protein effectively attenuates ischemic brain damage, highlighting its therapeutic promise for ischemic brain injury.

## Author Contributions

Z.J. and CSL designed the study, collected and analyzed data, interpreted the results, and prepared the initial manuscript draft. X.Q., L.J.Y., ZXM., and LP assisted with data acquisition and materials preparation, and critically reviewed the manuscript. HDZ. and J.L. contributed to study design and manuscript revision. All authors approved the final manuscript and assumed responsibility for ensuring the accuracy, transparency, and integrity of the research.

## Funding

We thank all individuals who contributed to this study. This work was supported by the National Natural Science Foundation of China (82271416), the Shandong Provincial Natural Science Foundation (ZR2022MH083, ZR2021MH226).

## Ethics Statement

All animal procedures were conducted under institutional ethical standards and received approval from the Ocean University of China Animal Care and Use Committee (Approval No. OUC‐AE‐2022‐152).

## Conflicts of Interest

The authors declare no conflicts of interest.

## Data Availability

The data that support the findings of this study are available from the corresponding author upon reasonable request.
